# Dr. Edward H. Tweedy

**Published:** 1908-06

**Authors:** 


Dr. Edward H. Tweedy, of Buffalo, died at his home May 13, 1908, aged 43 years. He became infected from a prick in his finger during a surgical procedure, which occurred about a month before his death. The infection proceeded from bad to worse notwithstanding prompt and continuous skilful treatment. He graduated from the medical department of the University of Buffalo in 1888, and has since practised his profession in this city. Dr. Tweedy is survived by his wife, his daughter Marion, his mother, two brothers and one sister, his father having died a few days after his own decease.

